# Oxalic acid application method and treatment intervals for reduction of *Varroa destructor* (Mesostigmata: Varroidae) populations in *Apis mellifera* (Hymenoptera: Apidae) colonies

**DOI:** 10.1093/jisesa/iead086

**Published:** 2023-12-06

**Authors:** Cody Prouty, Hossam F Abou-Shaara, Branden Stanford, James D Ellis, Cameron Jack

**Affiliations:** Entomology and Nematology Department, University of Florida, Gainesville, FL, USA; Entomology and Nematology Department, University of Florida, Gainesville, FL, USA; Department of Plant Protection, Faculty of Agriculture, Damanhour University, Damanhour, Egypt; Entomology and Nematology Department, University of Florida, Gainesville, FL, USA; Florida Department of Agriculture and Consumer Services, Division of Plant Industry, Bureau of Plant and Apiary Inspection, Gainesville, FL, USA; Entomology and Nematology Department, University of Florida, Gainesville, FL, USA; Entomology and Nematology Department, University of Florida, Gainesville, FL, USA

**Keywords:** *Varroa destructor*, *Apis mellifera*, oxalic acid, insect fogger, vaporization

## Abstract

Oxalic acid (OA) is a popular miticide used to control *Varroa destructor* (Mesostigmata: Varroidae) in western honey bee (*Apis mellifera* L.) (Hymenoptera: Apidae) colonies. Our aim was to investigate which method of OA application (dribbling, fogging, or vaporizing) was the most effective at reducing *V. destructor* infestations (Experiment 1) and to improve upon this method by determining the treatment interval that resulted in the greatest *V. destructor* control (Experiment 2). We used the product Api-Bioxal (97% OA) and maintained 40 honey bee colonies (10/treatment) in both experiments. In Experiment 1, the treatments included (i) dribbling 50 ml of 3% OA solution, (ii) vaporizing 4 g of solid OA, (iii) using an insect fogger supplied with 2.5% OA dissolved in ethyl alcohol, and (iv) an untreated control. After 3 weeks, only the vaporization method reduced *V. destructor* infestations (from 9.24 mites/100 bees pretreatment to 3.25 mites/100 bees posttreatment) and resulted in significantly increased brood amounts and numbers of adult bees over those of the controls. In Experiment 2, all colonies were treated with 4 applications of OA via vaporization at a constant concentration of 4 g OA/colony. In this experiment, the groups were separated by treatment intervals at either 3-, 5-, or 7-day intervals. We observed that 5- and 7-day treatment intervals significantly reduced *V. destructor* populations from pretreatment levels over that of the controls and 3-day intervals. Our data demonstrate the efficacy of OA in reducing *V. destructor* infestation, particularly vaporizing 4 g every 5–7 days as the most effective method of application.

## Introduction

The western honey bee, *Apis mellifera* L., is an economically important pollinator in agricultural systems and faces stressors such as pathogens, parasites, and pests (Boncristiani et al. 2021). Of these, the ectoparasitic mite, *Varroa destructor*, is the most devastating pest honey bees encounter*. Varroa destructor* is an important vector of pathogens, such as deformed wing virus (Martin and Brettell 2019), and correlates strongly with colony losses (Highfield et al. 2009). As a result, beekeepers globally treat colonies to maintain low mite infestations, as untreated colonies often do not last a single year without treatment (Jacques et al. 2017, Jack et al. 2023). Despite the availability of different control options developed to reduce *V. destructor* populations in honey bee colonies, beekeepers typically rely most on chemical treatments due to their high efficacy and ease of use (Woodford et al. 2023). Unfortunately, the mite development of resistance to synthetic miticides has become a significant issue in the beekeeping industry (Elzen and Westervelt 2002, González-Cabrera et al. 2016, Rinkevich et al. 2023).

Oxalic acid (OA) is a natural chemical that has been shown to reduce the incidence of *V. destructor* within bee colonies, while maintaining safety for bees (e.g., Popov et al. 1989). In the United States, Api-Bioxal (97% OA) is the current labeled OA treatment for *V. destructor*. There have been no reports of resistance of *V. destructor* to OA to date (Maggi et al. 2016b). There are a few methods commonly used to treat colonies with OA. The “trickling” or “dribble” method involves pouring a sugar water/OA mixture between frames over the adult honey bees. This is the traditional application method for OA and has been used for decades in several European countries (Rademacher and Harz 2006). It has a long history of use in North America as well (Aliano and Ellis 2008). Although trickling methods are the most widely used methods to administer OA to colonies (Charriére and Imdorf 2002, Rademacher and Harz 2006, Maggi et al. 2016a), vaporization is becoming increasingly popular (Gregorc and Sampson 2019, Jack et al. 2021, Evans et al. 2022, Kanelis et al. 2023). In the United States, several investigations have consistently demonstrated the inefficacy of OA vaporization at the current labeled rate of 1 g OA/brood chamber, but have shown an improved efficacy at 2–4 g of OA administered per brood chamber (Jack et al. 2021, Berry et al. 2022). Furthermore, some studies have produced contradicting results regarding which method of OA application is most effective at controlling *V. destructor* (Al Toufailia et al. 2015, Gregorc et al. 2016). Current trends in *V. destructor* control methods and beekeeper adoption, particularly economic barriers and development of products, were recently summarized in Bartlett (2022).

Recently, beekeepers in the United States have reported using insect foggers to administer OA to honey bee colonies. Although this is not a labeled application method for OA, it has been touted as a quicker and cheaper delivery system than vaporization. Fogging, as a method, has been shown to increase honey bee mortality when delivering thyme oil (Abou-Shaara et al. 2022), though the effect of its delivery of OA has not been accurately measured. Fogging chemicals into bee hives could negatively impact the applicator, given that many insect foggers work by igniting propane to burn chemicals mixed with ethyl alcohol. In certain circumstances, there is potential for the ignition of gas or alcohol outside of the system, unless using electric insect foggers.

OA applications are only effective at killing *V. destructor* that are outside of brood cells, either feeding on adult honey bee fat bodies or moving to a new cell (Gregorc and Planinc 2001). OA has also been shown to be toxic to bee larvae (Terpin et al. 2019). Therefore, the interval that treatments are applied is extremely important, and timing plays a major role. Female mites enter brood cells containing fifth-instar bee larvae (Piou et al. 2016). There, they hide in bee bread until other adult bees cap the cells, then feed on prepupa, and lay eggs on the developing bee (Traynor et al. 2020). Juvenile mites hatch from the eggs, develop, mate, and then exit the cell when the parasitized bee ecloses. Female *V. destructor* require 7–8 days to develop in capped brood cells (Rosenkranz et al. 2010). Given this life cycle, *V. destructor* are only exposed to treatments prior to entering cells and once adult bees eclose. Consequently, mites are safe within cells for ~10–15 days each reproductive cycle, depending on whether they invade worker or drone brood cells. In the studies we present here, minimal drone brood was present due to the season in which experiments were conducted.

Here, we aimed to determine (i) the most effective method (trickling, fogging, or vaporizing) for administering OA to honey bee colonies and (ii) the ideal treatment interval (3, 5, or 7 days) for vaporizing OA. Given that OA does not penetrate brood cells, developing *V. destructor* are not exposed to treatments. Therefore, we hypothesized that treatments administered more frequently than every 7 days would be less effective than those administered at 7-day intervals. The 7-day interval would expose honey bees eclosing from cells to OA, correspondingly targeting emerging *V. destructor* as well as those already on adult bees. More frequent application intervals would lead to treatment completion before all capped bees eventually emerge, thus being unavailable to mites developing in capped brood cells. Based on previous research on OA administration and dosing (Jack et al. 2021, Abou-Shaara et al. 2022), we further hypothesized that vaporizing OA at an elevated dose (i.e., over the currently labeled rate) would be the most effective treatment application, while administering OA with an insect fogger could impact colony health negatively. Finally, we hypothesized that vaporizing OA would be more effective at controlling *V. destructor* than fogging and dribbling the OA.

## Materials and Methods

### Experiment 1—Optimal Application Method

We used 40 colonies of European-derived honey bee stock at the University of Florida’s Plant Science Research and Education Unit (PSREU) in Citra, FL, USA (29.41°N, 82.17°W) during late February–March 2022. At the beginning of the study, all colonies were queenright and contained brood. *Varroa destructor* were confirmed to be present in all experimental colonies, and each colony was established in 10-frame Langstroth hives, consisting of a single deep hive body, a migratory lid, a solid bottom board, an entrance reducer, and a feeder jar placed on the lid. The colonies were managed on 4-way pallets and according to standard best practices for the region and the season. This included feeding the colonies weekly with a 1:1 sugar syrup to ensure they would not starve due to a lack of resources. Colonies were equalized prior to study initiation to ensure each hive contained 3–4 frames of brood and 3–4 frames of resources (combination of honey, nectar, and/or pollen). After equalization, no combs were shared between colonies to ensure no further mechanical transfer of *V. destructor*. Each colony was randomly assigned to one of 4 treatment groups such that there were 10 colonies per group. Once sorted into 4 groups, a random number generator was used to assign each group a treatment interval.

#### OA exposure treatments.

We used the commercially available product Api-Bioxal (Chemicals Laif, Italy, Vigonza; OA, 97.0%) for all colonies receiving an OA treatment. OA was applied in the colonies using 3 methods: (i) vaporizing 4 g per colony using an OA vaporizer (ProVap110 vaporizer [OxaVap, Manning, SC, USA]), (ii) trickling (dribble) of OA solution (3% or ~1.75 g OA/colony) using a 50-ml syringe, and (iii) using an insect fogger supplied with 2.5% OA concentration (25 g of OA dissolved in 100 ml of absolute ethyl alcohol). One puff lasting ~6 s was fogged per colony. The duration and concentration represent common practices described by beekeepers. To determine the amount of OA delivered using this method, we dissolved 25 g of OA into 100-ml ethyl alcohol. After 6 s, we measured the remaining volume of alcohol. After repeating this several times, the average volume delivered after 6 s was 2.96-ml solution (or 0.74 g of OA). The OA treatments were applied 3 times with 7-day intervals between each application. Colonies in the negative control group were not treated with OA.

#### Varroa destructor infestation rates.

*Varroa destructor* infestation rates were determined using alcohol washes (95% ethanol) with an estimated 100 bees per sample. Samples were collected from frames containing emerging adult honey bee workers 3 days before the start of the project and 1 week after the last treatment application (Dietemann et al. 2013). We poured samples over sieves to separate mites from the bees, rinsed the bees with water, and then counted the mites and bees as described in Jack et al. (2020). Infestation rates were calculated from mite and bee counts (average number of mites/100 adult bees = infestation).

#### Seventy-two-hour mite fall.

We collected mite fall data for each group by placing sticky boards (Mann Lake, Hackensack, MN, USA; product: DC681) with mesh covering through the entrance and on the bottom of each hive for 72 h after each treatment. Following this, we collected the sticky boards and counted the number of mites on the boards accumulated during the 72 h.

#### Colony evaluations.

Colony evaluations were conducted visually to measure colony strength parameters such as adult bee populations (number of bees per colony, estimated by area) and brood estimates (cm^2^), as described by Delaplane et al. (2013). The evaluations were completed twice: once 3 days prior to treatment and again 1 week after the final application of the treatments. The same individual performed both evaluations to standardize the estimates as much as possible.

#### Dead bees in front of the hives.

We counted dead bees in front of the hives 24 h after each treatment application. We laid inverted telescoping covers in front of each hive entrance immediately following each treatment application to catch dead bees that were removed from colonies by undertaker bees. After 24 h, the number of dead bees found in these lids was quantified. This method was repeated after each OA application.

### Experiment 2—Vaporization Interval

We used 40 colonies of European-derived honey bee stock at the University of Florida’s Beef Research Unit (UFBRU) in Gainesville, FL, USA (29.74°N, 82.26°W) during November–December 2021.

#### OA treatments.

We used Api-Bioxal (Chemicals Laif, Vigonza, Italy; OA, 97.0%) for all treatments. Four grams of OA were applied to the 3 OA-treated colony groups via vaporization using the. Each group was treated 4 times, and we staggered the start date for each group to ensure all treatments would be completed the same week (Supplementary Table S1). The interval timing included (i) 3-day intervals, (ii) 5-day intervals, and (iii) 7-day intervals. A fourth, untreated group of colonies served as a negative control group.

#### Varroa destructor infestation rates.

*Varroa destructor* infestation rates were determined using alcohol washes as described for Experiment 1.

#### Seventy-two-hour mite fall.

We collected mite fall data for each treatment group using the method described for Experiment 1.

### Statistical Analysis

All data were analyzed using R version 4.1.1. In Experiment 1, colony evaluations (estimations of bee numbers and cm^2^ brood), dead bee counts, mites/100 bees (alcohol washes), and the 72-hour mite fall were analyzed using linear mixed models with normal error distributions in the lme4 package. Residuals were checked using the DHARMa package in R. Both variables in Experiment 2 (mites/100 bees and 72-hour mite fall) were analyzed using generalized linear mixed models with negative binomial distributions (θ = 2) in the lme4 package (Bates et al. 2015). Residuals were again checked using the DHARMa package in R (Hartig and Hartig 2017). In both experiments, we tested the interaction between treatment and time, with colony number as a random effect. When interactions between the two were significant, we compared dependent variable responses separately using a 1-way analysis of variance (ANOVA), subset by treatment for variables that were measured pre- and postapplication and time for variables measured at each application based on our hypotheses for each response variable. Additionally, in both experiments, we calculated the difference of *V. destructor* infestation from pre- to posttreatment. This variable was analyzed using a 1-way ANOVA (normal distribution) by treatment. Post hoc tests in both experiments were performed using Tukey’s honestly significant difference.

## Results

### Experiment 1—Optimal Application Method

#### Colony evaluations.

**Fig. 1.**
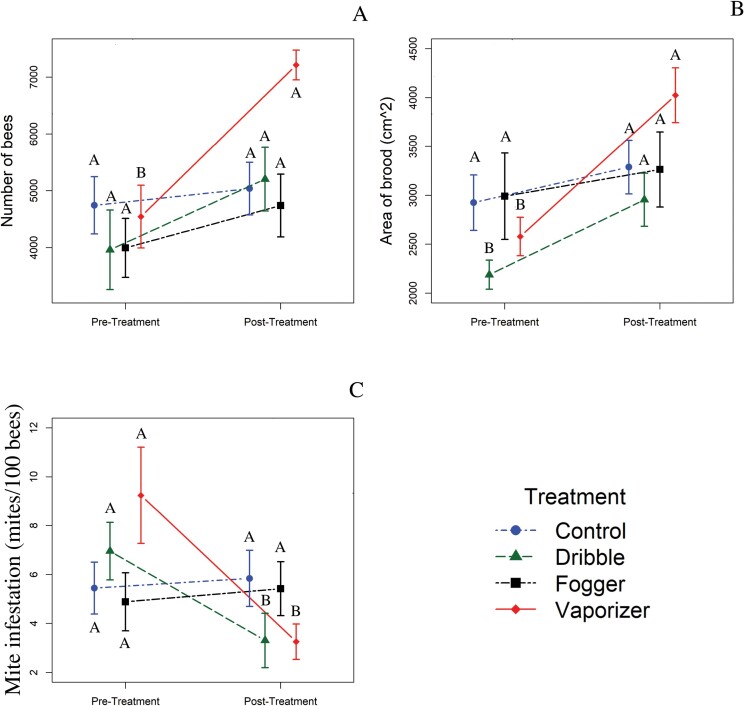
Experiment 1. A) Number of adult honey bees as estimated by colony evaluations. B) Area of brood (cm^2^) as estimated by colony evaluations. C) *Varroa destructor* infestation rates (number of mites/100 bees) pre- and postapplication with OA. The points are means, while the bars are standard errors. Means with different letters are different at α ≤ 0.05. Comparisons are made within treatment (i.e., between the pre- and posttreatment means for each treatment).

There was a significant interaction between treatment and time on the average number of bees and estimated brood area in the colonies (Table 1). When analyzed by treatment, colonies treated with OA via vaporization had significantly more bees and brood postapplication than preapplication (Fig. 1A and B; Table 2). Colonies treated with OA via dribble had significantly more brood postapplication than preapplication (Fig. 1B), but no differences existed between the number of bees (Table 2). These trends did not exist in untreated control colonies or those treated with OA via fogging (Table 2).

**Table 1.** Main effects of the results in experiments 1 and 2. All response variables were analyzed with treatment, time, and the interaction between the two as fixed effects and with the colony as a random effect. Significant results are in bold font.

Response variable	Predictor variable	df	*F*-value	*P*-value
Experiment 1				
Number of bees	Treatment	3	1.88	0.151
	**Time**	**1**	**20.06**	**<0.001**
	**Treatment × time**	**3**	**4**	**0.016**
Area of brood	Treatment	3	1.23	0.313
	**Time**	**1**	**31.21**	**<0.001**
	**Treatment × time**	**3**	**6.78**	**0.001**
Dead bees at hive entrance	Treatment	3	0.31	0.82
	Time	2	2.12	0.127
	Treatment × time	6	0.13	0.993
Seventy-two-hour mite fall	**Treatment**	**3**	**25.06**	**<0.001**
	**Time**	**2**	**19.68**	**<0.001**
	**Treatment × time**	**6**	**6.88**	**<0.001**
*Varroa destructor* infestation rates	Treatment	3	0.39	0.76
	**Time**	**1**	**8.49**	**0.006**
	**Treatment × time**	**3**	**4.41**	**0.01**
Difference in *V. destructor* infestation rates	**Treatment**	**3**	**4.55**	**0.009**
Experiment 2				
Seventy-two-hour mite fall	**Treatment**	**3**	**5.22**	**0.004**
	**Time**	**3**	**31.44**	**<0.001**
	**Treatment × time**	**9**	**5.55**	**<0.001**
*Varroa destructor* infestation rates	Treatment	3	1.67	0.19
	**Time**	**1**	**14.49**	**<0.001**
	**Treatment × time**	**3**	**5.8**	**0.003**
Difference in *V. destructor* infestation rates	**Treatment**	**3**	**6.23**	**0.002**

**Table 2.** For significant interaction variables in Table 1, response variables were subset by one predictor variable and analyzed by the second as described in the Statistical analysis section of the Methods. Significant results are in bold font.

Response variable	Predictor variable 1	Predictor variable 2	df	*F*-value	*P*-value
Experiment 1					
Number of bees	Treatment: control	Time	1	0.18	0.673
	**Vaporizer**	**Time**	**1**	**17.72**	**<0.001**
	Dribble	Time	1	1.78	0.2
	Fogger	Time	1	0.97	0.339
Area of brood	Treatment: control	Time	1	0.85	0.369
	**Vaporizer**	**Time**	**1**	**18.48**	**<0.001**
	**Dribble**	**Time**	**1**	**6.81**	**0.019**
	Fogger	Time	1	0.21	0.65
Dead bees at hive entrance	Time: 1	Treatment	3	0.17	0.915
	Time: 2	Treatment	3	0.36	0.785
	Time: 3	Treatment	3	0.05	0.983
Seventy-two-hour mite fall	**Time: 1**	**Treatment**	**3**	**11.33**	**<0.001**
	**Time: 2**	**Treatment**	**3**	**21.15**	**<0.001**
	**Time: 3**	**Treatment**	**3**	**8.96**	**<0.001**
*Varroa destructor* infestation rates	Treatment: control	Time	1	0.06	0.803
	**Vaporizer**	**Time**	**1**	**7.44**	**0.014**
	**Dribble**	**Time**	**1**	**5.03**	**0.039**
	Fogger	Time	1	0.11	0.747
Experiment 2					
Seventy-two-hour mite fall	**Time: 1**	**Treatment**	**3**	**6.11**	**0.002**
	**Time: 2**	**Treatment**	**3**	**4.65**	**0.008**
	**Time: 3**	**Treatment**	**3**	**3.09**	**0.039**
	Time: 4	Treatment	3	0.07	0.977
*Varroa destructor* infestation rates	Interval: control	Time	1	0.26	0.618
	3 day	Time	1	0.87	0.364
	**5 day**	**Time**	**1**	**14.11**	**0.002**
	**7 day**	**Time**	**1**	**13.46**	**0.002**

#### Dead bees at hive entrance.

**Fig. 2.**
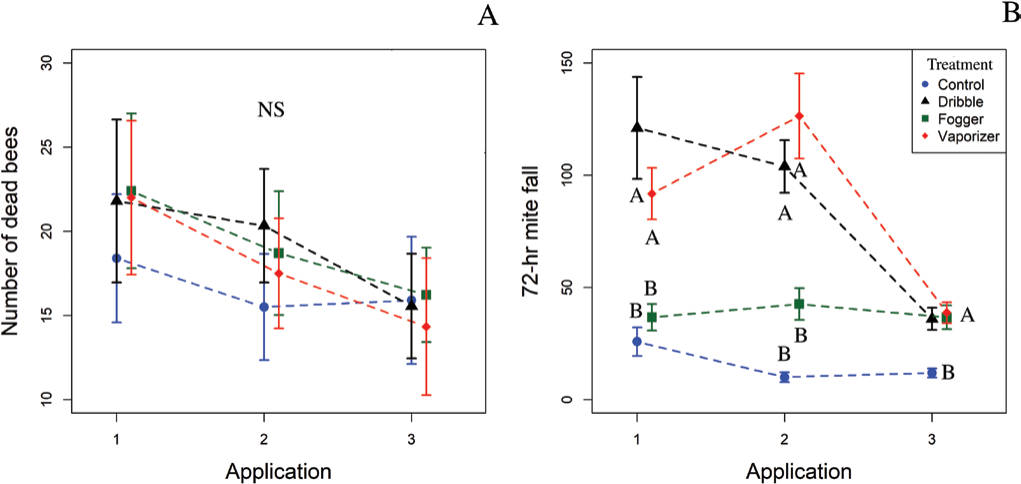
Experiment 1. A) Number of dead adult honey bees counted at the hive entrance. B) Seventy-two-hour mite fall at each treatment application. Means with different letters within a sampling time are significantly different at α ≤ 0.05. Comparisons are made within the OA application (i.e., between the application method means for each sampling time). Points are means and bars represent standard error. NS = no significant differences found.

There was no significant effect of treatment, time, or the interaction between treatment and time on the number of dead bees counted at the hive entrance (Table 1; Fig. 2A).

#### Seventy-two-hour mite fall.

There was significant treatment × time interaction on the number of *V. destructor* recovered from 72-hour mite fall (Table 1). When analyzed by application, there was significant differences in 72-h mite fall between treatments in all applications (Table 2). The dribble and vaporizer treatments had significantly greater mite fall than the control and fogger treatments for applications 1 and 2. In application 3, the dribble, vaporizer, and fogger treatments had significantly greater mite fall than the control (Fig. 2B).

#### Varroa destructor infestation rates.

**Fig. 3.**
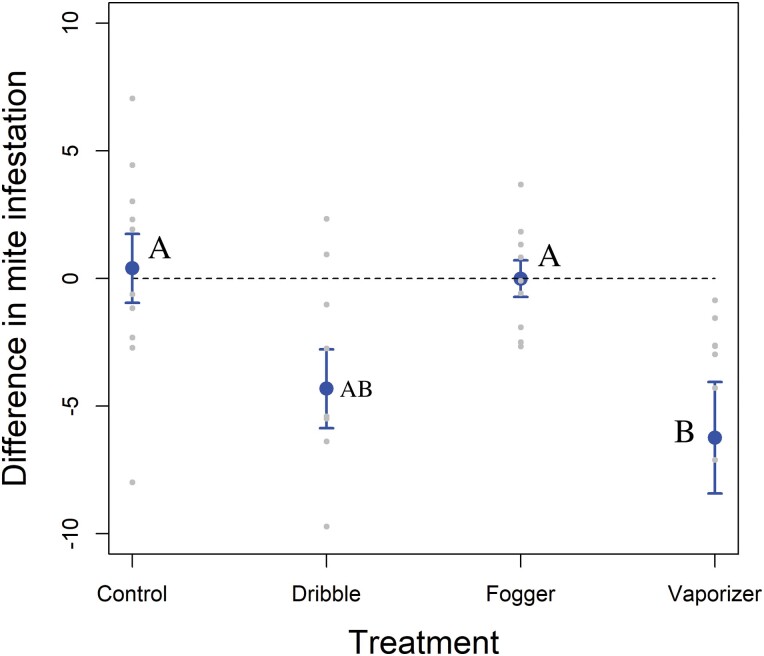
Experiment 1. Difference in *Varroa destructor* infestation rates (number of mites/100 bees) from pre- to postapplication in Experiment 1. Points are means, and bars are standard errors. Jittered points are individual colonies. The dashed line represents no change.

There was a significant interaction between treatment and time for mite infestation rates (Table 1). When analyzed by treatment, significantly fewer mites were present postapplication than preapplication for the vaporization and dribble (Table 2; Fig. 1C) treatments. There was a significant effect of the difference in *V. destructor* infestation between treatments for the pre- and post-applications. Colonies treated with OA administered via vaporizer had greater reduction in mite infestations than did colonies in the control group. Colonies treated via dribble did not differ from any other treatment (Table 2; Fig. 3).

### Experiment 2—Vaporization Interval

#### Seventy-two-hour mite fall.

**Fig. 4.**
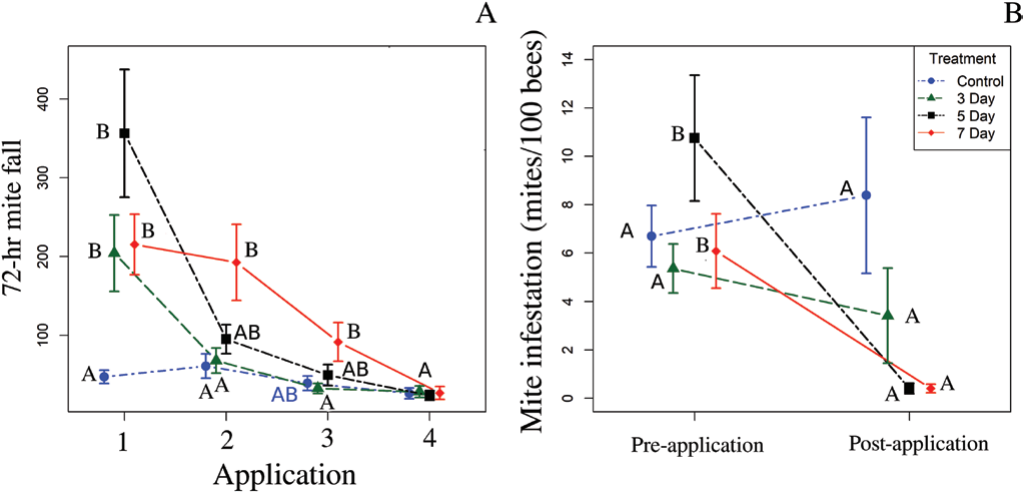
Experiment 2. A) Average 72-hour mite fall at each OA application. B) *Varroa destructor* infestation rates (number of mites/100 bees) pre- and postapplication with OA. Means with different letters within a sampling time are significantly different at α ≤ 0.05. Comparisons are made within the OA application for (A) and within the treatment for (B). Points are means, and bars represent standard error.

There was a significant interaction between treatment interval and time sampled for 72-h mite fall (Table 1), so this parameter was analyzed by time sampled. After the first OA application, 72-h mite fall in colonies in the 3-, 5-, and 7-day interval groups was significantly higher than that in the control group (Table 2). After the second application, 72-h mite fall in the 7-day interval group was higher than that in the 3-day interval and control groups. The 72-h mite fall in the 5-day interval group did not differ from those of the other groups. After the third application, 72-h mite fall differed between the 7-day interval group and 3-day interval group, but not between any other group combinations (Table 2). All groups had statistically similar 72-h mite falls after the fourth OA application (Fig. 4A).

#### Varroa destructor infestation rates.

**Fig. 5.**
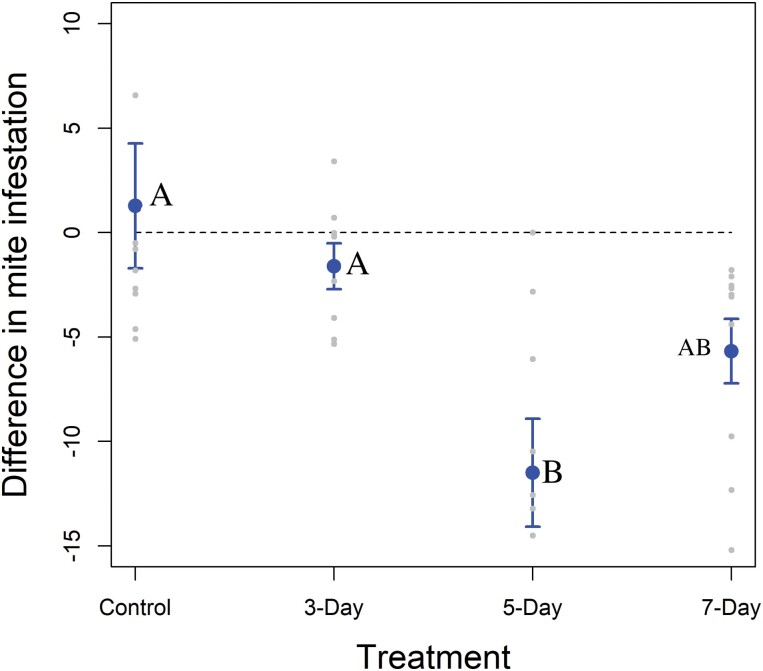
Experiment 2. Difference in *Varroa destructor* infestation rates (number of mites/100 bees) from pre- to postapplication in Experiment 2. Points are means, and bars are standard errors. Jittered points are individual colonies. The dashed line represents no change.

There was a significant interaction between treatment interval and time sampled on *V. destructor* infestation rates (Table 1), so this parameter was analyzed by treatment. Controls and the 3-day interval groups did not differ from pre- to postapplication (Table 2; Fig. 5). *Varroa destructor* infestations rates were significantly lower postapplication than preapplication for both 5- and 7-day intervals (Table 2; Fig. 4B).

## Discussion

The insect fogger treatment as currently applied by many beekeepers was shown to be an ineffective delivery method for OA in a mite control capacity. We estimated that the fogger delivers only ~0.74 g of OA to each colony when following common beekeeper application protocols. This contrasts with the 4 and 1.75 g administered per colony using the vaporizer and dribble methods, respectively. The insect fogger is less precise than the vaporizer, and this limits the ability of an applicator to estimate the amount of OA delivered to the colony accurately at the time of application. Although there is evidence in the literature that vaporization of OA is effective against *V. destructor* and safe for honey bees (Gregorc and Planinc 2012), there is currently no research on whether the same is true when OA is applied using an insect fogger. Some research on using thyme oil applied via an insect fogger exists; however, the researchers observed an increase in honey bee mortality when administering thyme oil this way (Abou-Shaara et al. 2022). Based on our results, we did not see evidence that fogging OA negatively impacted honey bee health, but there was also no evidence that it reduced *V. destructor* infestation. We conclude that applying OA using insect foggers may not reduce *V. destructor* populations effectively. More research is needed to determine whether this is always the case.

Interestingly, the difference between the OA dose in fogging and dribble is close to the difference in OA dose between dribble and vaporization of 4 g. This observation indicates that the efficacy of OA treatments is likely due to a combination of 2 factors: dose and dispersal. Fogging OA and vaporizing OA have a similar dispersal, but the doses between these methods in this experiment were widely different. Dribbling OA has a limited dispersal and does not reach as many bees and mites. Although we observed a reduction in mite infestations, dribbling OA did not provide us with reductions that were statistically significant from pretreatment levels, varying from other researchers that have observed significant reductions with this method and dose (reviewed by Gregorc and Sampson 2019). As OA does not penetrate brood cappings and is less effective while brood is present (Gregorc and Planinc 2001), it is likely that the current labeled rate used for dribbling OA was insufficient for reducing *V. destructor* populations under the colony conditions in this experiment.

Vaporization of OA also has limitations. For example, 1 g of OA (the labeled rate) delivered via vaporization has shown mixed impacts on mite loads (Rademacher and Harz 2006, Gregorc et al. 2016, Jack et al. 2020, Berry et al. 2022). Ultimately, it is difficult to compare beekeeper use patterns of fogging and vaporizing OA given the different amounts of OA delivered per colony using the 2 different application methods. Based on our results, vaporizing and dribbling are equally effective in increasing 72-h mite fall, but colonies treated with OA via vaporization had the greatest number of bees and area of brood. Therefore, in terms of overall colony health, vaporizing OA at a 4-g dose is the ideal method of application for *V. destructor* treatment, though dribbling is an adequate alternative if vaporization equipment is not available.

The differing 72-h mite fall patterns seen in Experiment 2 between the 3 interval groups are worth discussing. For example, colonies treated with OA every 3 days had similar mite falls to those treated with OA every 5 and 7 days at the first time point, but not at the subsequent ones. We suspect that this treatment killed the mites outside of brood cells at the first treatment but missed those developing and safe in brood cells upon successive 3-day treatments. Ultimately, we feel that a 5- or 7-day treatment interval is optimum for vaporizing OA as it allows for more mites to be present on adult bees, and vulnerable to OA treatment, given the extended application period. We hypothesize that shorter treatment intervals, such as those below 5 days, do not effectively reach *V. destructor* that are outside of brood cells.

Jack et al. (2020) and Berry et al. (2022) found that the label rate of 1 g OA/brood chamber was ineffective at reducing mite populations, but that a higher dose of 2–4 g could significantly reduce mite populations. Based on these findings, experiments in the present study were performed using a 4 g/brood chamber, since this rate was not shown to have a detrimental effect on honey bee health (Jack et al. 2021). Berry et al. (2022) also found no significant changes in the adult bees, brood, or stored honey when colonies were treated with vaporized OA, suggesting safety to bees when this delivery method is used.

When treating colonies with OA, we recommend using vaporization to deliver OA to colonies. Furthermore, we recommend increasing the treatment intervals for vaporization to every 7 days, ideally, as 3-day intervals are too frequent. Ultimately, we conclude that vaporizing 4 g of solid OA at 7-day treatment intervals is the most efficient way to apply OA to honey bee colonies for controlling *V. destructor*. Before this treatment protocol could become available to U.S. beekeepers, the Api-Bioxal label would need to be amended to allow for a higher dose of OA per brood chamber. Given our observations of OA treatment effects on honey bee colonies, we would be in favor of such a label change. Additional work should elucidate how many applications of OA vaporization are needed to reduce *V. destructor* populations below damaging thresholds effectively and how long these treatment regimens will protect honey bee colonies.

## Supplementary Material

iead086_suppl_Supplementary_Tables_S1
