# Supplementary material for: Oxalic acid application method and treatment intervals for reduction of Varroa destructor (Mesostigmata: Varroidae) populations in Apis mellifera (Hymenoptera: Apidae) colonies
Source: J Insect Sci. 2023 Dec 6;23(6):13. doi: 10.1093/jisesa/iead086 (PMC10699871; doi:10.1093/jisesa/iead086)
Supplement: iead086_suppl_Supplementary_Tables_S1 [file iead086_suppl_supplementary_tables_s1.docx]

Table S1: Schedule for treatments and sampling, experiment 2.

| **Time period** | **Time relative to start of experiment** | **Description of events** |
| --- | --- | --- |
| 1 | day 0 | Baseline data were collected for *Varroa destructor* levels in all colonies. 7-day interval colonies treated with 4 g OA. Sticky boards placed in control colonies and 7-day colonies. |
| 2 | day 7 | 7-day interval colonies treated with 4 g OA. Sticky boards placed in control colonies and 7-day colonies. |
| 3 | day 8 | 5-day interval colonies treated with 4 g OA. Sticky boards placed in 5-day interval colonies. |
| 4 | day 10 | Removed sticky boards that were placed in the control and 7-day colonies |
| 5 | day 11 | Removed sticky boards that were placed in the 5-day colonies |
| 6 | day 13 | 5-day interval colonies treated with 4 g OA. Sticky boards placed in 5-day interval colonies. |
| 7 | day 14 | 7-day interval colonies treated with 4 g OA. Sticky boards placed in control colonies and 7-day colonies. |
| 8 | day 16 | Removed sticky boards that were placed in the 5-day colonies. 3-day interval colonies treated with 4 g OA and sticky boards were placed. |
| 9 | day 17 | Removed sticky boards that were placed in the control and 7-day colonies. |
| 10 | day 18 | 5-day interval colonies treated with 4 g OA. Sticky boards placed in 5-day interval colonies. |
| 11 | day 19 | Removed sticky boards that were placed in the 3-day colonies. 3-day interval colonies treated with 4 g OA and sticky boards were placed. |
| 12 | day 21 | 7-day interval colonies treated with 4 g OA. Sticky boards placed in control colonies and 7-day colonies. Removed sticky boars that were placed in the 5-day colonies. |
| 13 | day 22 | Removed sticky boards that were placed in the 3-day colonies. 3-day interval colonies treated with 4 g OA and sticky boards were placed. |
| 14 | day 23 | 5-day interval colonies treated with 4 g OA. Sticky boards placed in 5-day interval colonies. |
| 15 | day 24 | Removed sticky boards that were placed in the control and 7-day colonies. |
| 16 | day 25 | Removed sticky boards that were placed in the 3-day colonies. 3-day interval colonies treated with 4 g OA and sticky boards were placed. |
| 17 | day 26 | Removed sticky boards that were placed in the 5-day colonies. |
| 18 | day 28 | *Varroa destructor* levels were measured for all colonies and colony mortality was noted. |

Table S2: Honey bee colony strength estimates and *Varroa destructor* infestations pre- and post-oxalic acid treatment administration in Experiment 1. Data are mean ± SE (N). Letters represent significance at the 0.05 alpha level. Comparisons were made between pre- and post-treatment means within each treatment.

| Treatment | Sampling time | Number of adult honey bees | Cm^2^ brood | *Varroa* *V. destructor* infestation (# mites per /100 bees) |
| --- | --- | --- | --- | --- |
| Control | Pre-application | 4,743.78 ± 503.98 (10) a | 2,924.8 ± 283.28 (10) a | 5.44 ± 1.06 (10) a |
|  | Post-application | 5,037.37 ± 461.62 (10) a | 3,288.75 ± 274.86 (10) a | 5.84 ± 1.15 (10) a |
| Dribble | Pre- application | 3,961.31 ± 698.2 (10) a | 2,188.12 ± 148.7 (10) b | 6.95 ± 1.18 (10) b |
|  | Post- application | 5,202.82 ± 559.55 (8) a | 2,954.4 ± 271.95 (8) a | 3.3 ± 1.11 (9) a |
| Fogger | Pre- application | 3,996.14 ± 518.52 (10) a | 2,990.57 ± 442.23 (10) a | 4.88 ± 1.19 (10) a |
|  | Post- application | 4,739.78 ± 551.2 (9) a | 3,264.39 ± 384.72 (9) a | 5.42 ± 1.1 (9) a |
| Vaporizer | Pre- application | 4,544.74 ± 552.26 (10) b | 2,578.38 ± 195.97 (10) b | 9.24 ± 1.97 (10) b |
|  | Post- application | 7,213.95 ± 261.64 (9) a | 4,024.46 ± 280.28 (9) a | 3.25 ± 0.72 (9) a |

Table S3: Counts of dead adult honey bees and *Varroa destructor* on sticky boards at each sampling event in Experiment 1. Data are mean ± SE (N). Letters represent significance at the 0.05 alpha level. *

| Treatment | Sampling time | Dead bee counts | 72-hour mite fall |
| --- | --- | --- | --- |
| Control | Application 1 | 18.4 ± 3.81 (10) | 25.8 ± 6.35 (10) b |
|  | Application 2 | 15.5 ± 3.15 (10) | 10 ± 2.23 (10) b |
|  | Application 3 | 15.9 ± 3.78 (10) | 11.9 ± 2.04 (10) b |
| Dribble | Application 1 | 21.8 ± 4.84 (10) | 121.1 ± 22.73 (10) a |
|  | Application 2 | 20.33 ± 3.37 (9) | 103.89 ± 11.76 (9) a |
|  | Application 3 | 15.56 ± 3.11 (9) | 36 ± 4.96 (9) a |
| Fogger | Application 1 | 22.4 ± 4.6 (10) | 36.7 ± 5.91 (10) b |
|  | Application 2 | 18.7 ± 3.68 (10) | 42.6 ± 7.07 (10) b |
|  | Application 3 | 16.22 ± 2.81 (9) | 36.67 ± 5.34 (9) a |
| Vaporizer | Application 1 | 22 ± 4.58 (10) | 91.8 ± 11.46 (10) a |
|  | Application 2 | 17.5 ± 3.27 (10) | 126.4 ± 18.96 (10) a |
|  | Application 3 | 14.33 ± 4.07 (9) | 38.78 ± 4.66 (9) a |

Table S4: *Varroa destructor* infestations pre- and post-oxalic acid treatment administration in Experiment 2. Data are mean ± SE (N). Letters represent significance at the 0.05 alpha level. Comparisons were made between pre- and post-treatment means within each treatment.

| Treatment  Interval | Sampling time | *Varroa* *V. destructor* infestation (# mites per /100 bees) |
| --- | --- | --- |
| Control | Pre-application | 6.7 ± 1.27 (10) a |
|  | Post-application | 8.39 ± 3.22 (9) a |
| 3-Day | Pre- application | 5.36 ± 1.01 (10) a |
|  | Post- application | 3.41 ± 1.97 (8) a |
| 5-Day | Pre- application | 10.76 ± 2.6 (10) a |
|  | Post- application | 0.4 ± 0.22 (9) b |
| 7-Day | Pre- application | 6.08 ± 1.54 (10) a |
|  | Post- application | 0.4 ± 0.18 (10) b |

Table S5: Counts of *Varroa destructor* on sticky boards at each sampling event in Experiment 2. Data are mean ± SE (N). Letters represent significance at the 0.05 alpha level. *

| Treatment  Interval | Sampling time | 72-hour mite fall |
| --- | --- | --- |
| Control | Application 1 | 47.1 ± 8.26 (10) b |
|  | Application 2 | 60.8 ± 15.44 (10) b |
|  | Application 3 | 39 ± 9.18 (10) ab |
|  | Application 4 | 26.2 ± 6.87 (10) a |
| 3-Day | Application 1 | 204.2 ± 48.58 (10) a |
|  | Application 2 | 67.9 ± 15.92 (10) b |
|  | Application 3 | 32.5 ± 6.43 (10) b |
|  | Application 4 | 28.25 ± 7.25 (8) a |
| 5-Day | Application 1 | 356.4 ± 81.07 (10) a |
|  | Application 2 | 95 ± 18.31 (10) ab |
|  | Application 3 | 49.4 ± 13.41 (10) ab |
|  | Application 4 | 23.56 ± 6.02 (9) a |
| 7-Day | Application 1 | 215.3 ± 38.31 (10) a |
|  | Application 2 | 192.4 ± 48.4 (10) a |
|  | Application 3 | 91.4 ± 24.53 (10) a |
|  | Application 4 | 26.6 ± 8.21 (10) a |
